# Harnessing Mitochondrial Function for Post-Stroke Rehabilitation: Unlocking Antioxidant Power

**DOI:** 10.3390/antiox14091080

**Published:** 2025-09-03

**Authors:** Gabriela Olaru, Ana-Maria Buga, Raluca Elena Sandu, Vlad Padureanu, Dragos George Popa, Daniela Calina

**Affiliations:** 1 Doctoral School, University of Medicine and Pharmacy of Craiova, 200349 Craiova, Romania; 2 Department of Biochemistry, University of Medicine and Pharmacy of Craiova, 200349 Craiova, Romania; 3 Department of Internal Medicine, University of Medicine and Pharmacy of Craiova, 200349 Craiova, Romania; 4 Department of Plastic Surgery, University of Medicine and Pharmacy of Craiova, 200349 Craiova, Romania; 5 Department of Clinical Pharmacy, University of Medicine and Pharmacy of Craiova, 200349 Craiova, Romania

**Keywords:** antioxidant therapies, ischemic stroke, rehabilitation strategies, oxidative stress

## Abstract

Post-stroke rehabilitation has evolved to encompass advanced approaches that aim to optimize recovery for ischemic stroke survivors. Despite this progress, recovery remains limited, partly due to persistent oxidative stress and mitochondrial dysfunction that contribute to neuronal and muscular impairment. One such promising avenue is the stimulation of antioxidant capacity and the enhancement of mitochondrial function. Mitochondria are crucial for energy production and neuroprotection, which are essential for neurorecovery. This review explores the mechanisms involved in the role of mitochondrial function and antioxidant therapies, focusing on motor recovery after ischemic stroke and “brain-muscle axis” interplay in post-stroke rehabilitation. A comprehensive synthesis of clinical trial data is provided, highlighting interventions targeting mitochondrial bioenergetics, redox regulation, and mitochondrial dynamics. Furthermore, the review delves into the potential of recent mitochondrial-targeted therapies as adjuncts to traditional rehabilitation techniques, providing a more holistic approach to recovery. Emerging evidence suggests these therapies can reduce oxidative injury and support neuroplasticity; however, translation into consistent clinical benefit remains uncertain due to heterogeneity in study designs, endpoints, and patient populations. By understanding and leveraging the dynamics of mitochondrial function, healthcare providers can significantly enhance the rehabilitation outcomes for people with a range of conditions, from musculoskeletal disorders to neurological impairments.

## 1. Introduction

Stroke is one of the leading causes of long-term disability and mortality worldwide. Recent data indicates that approximately 11% of the global population dies because of stroke [1,2]. Moreover, stroke survivors and their families are affected by long-term disability due to limited recovery. Approximately 30% of stroke survivors require permanent care regarding daily activities [3]. Stroke incidence and prevalence in Europe is 1.1 million total cases per year; 80% of cases are accounted for ischemic stroke [3,4,4]. The incidence of stroke is estimated to increase in the next decade due to increased life expectancy and aging population. In addition, it is estimated that 1 in 4 stroke survivors will experience a new stroke in the next five years [5]. All of these will dramatically increase the healthcare costs due to the loss of productivity and healthcare expenses.

Despite improving public health politics and research efforts, the only available treatment for ischemic stroke remains intravenous thrombolysis (rtPA) and/or endovascular thrombectomy in the first 4.5 h from stroke onset [6,7,8,9,10]. This intervention is challenging, especially in low- and middle-income countries where only up to 16% of cases benefit from thrombolytic intervention. In this context, the critical needs are represented by enhanced stroke prevention strategies, on-time access to thrombolytic therapy, and effective post-stroke rehabilitation services.

Cerebral ischemia occurs due to the blood vessel obstruction that blocks the oxygen and nutrients delivery to the corresponding area. Restoring cerebral blood flow after ischemic stroke has a dual effect. On one hand, reperfusion is essential to salvage brain tissue, limit infarct expansion, and reduce long-term cognitive and functional deficits. On the other hand, the same process can paradoxically induce ischemia/reperfusion injury, particularly in the penumbral zone where neurons are still viable but highly vulnerable. Thus, reperfusion represents both a life-saving intervention and a potential source of secondary injury [7]. The main ischemia/reperfusion injuries are related to cytotoxicity due to increased glutamate levels, mitochondrial dysfunction-associated oxidative stress and inflammation [11]. The penumbra zone of the infarct area has received much attention in the last four decades regarding the increased potential for neurorecovery after cerebral ischemia [12].

The medical literature has undergone rapid development in terms of redox imbalance induced by ischemia reperfusion after stroke [13,14,15,16,17,18,19]. The “Code Stroke” concept, introduced by Dr. Camilo R. Gomez and colleagues in 1994, was designed to expedite in-hospital treatment for acute ischemic stroke patients by implementing a rapid-response system to minimize therapeutic delays and improve patient outcomes [20]. Starting from the “Code Stroke” concept of Camilo Gomez and colleagues [20], this review aimed to draw attention to Redox “Code Stroke” in rehabilitation, which highlights the impact of time, neuroprotection against oxidative stress and the impact on rehabilitation of people during the “living with stroke” journey. The main objective remains to stabilize the penumbra area and extend the therapeutic and effective rehabilitation window.

In this light, recent preclinical data has highlighted the pivotal role of oxidative stress and mitochondrial dysfunction in the post-stroke recovery, with mitochondria-targeted antioxidants emerging as particularly promising candidates. Despite its efficacy in preclinical studies, their clinical translation remains at an early stage. An explanation for this can be related to its limited clinical availability, incomplete safety profile, and lacking large-scale randomized controlled clinical trials to constrain their current applicability in post-stroke recovery. In contrast, general antioxidants are more widely studied and currently used in various clinical contexts. Therefore, the main objective of this review is to critically evaluate the role of general antioxidants vs. mitochondria-targeted therapy and mitochondria transplantation in post-stroke recovery, highlighting both their potential and their limitation, while situating these findings within the broader context of the ongoing effort to develop more targeted therapies. In addition, we aimed to review the current available clinical data using mitochondria-targeted antioxidants in post-stroke recovery as a translational step from laboratory research to clinical application. To achieve this objective, we searched for recent available clinical data in international databases (PubMed, Web of Science, Clinicaltrial.gov) using combinations of key words: “stroke”, “ischemic stroke”, “antioxidants”, “oxidative stress”, “motor recovery”, “rehabilitation”, “randomized trial”, “vitamin”, “supplement”, “endaravone”, “N-acetylcysteine”, “coenzyme Q10”, “melatonin”, “alpha-lipoic acid”, or “exercise”. By situating these findings within the broader context of ongoing efforts to develop more targeted neuroprotective therapies, this review highlights key gaps in current knowledge and identifies promising strategies for improving functional recovery after stroke.”

### 1.1. The Brain-Muscle Axis and Its Susceptibility to Oxidative Stress—Barriers to Functional Recovery

Going back in time around 2.5 billion years ago, at the origins of life on the Earth, the oxygen molecule is closely related to our understandings of life evolution since the “Great Oxygenation Event” occurred [1,2]. Cyanobacteria were the first organisms on the earth that generated oxygen through oxygenic photosynthesis, a process that split water and released molecular oxygen [3]. This discovery ultimately drove the Great Oxidation Event, enabling the evolution of aerobic life [4]. At the beginning, the increase in very reactive free oxygen radicals created a toxic environment due to oxidation of organic compounds (e.g., deoxyribonucleic acid—DNA, ribonucleic acid—RNA). The subsequent adaptation allowed for aerobic respiration and the evolution of multicellular forms of life [4]. Nowadays, this magic molecule is mandatory for complex organism life which uses oxygen to produce energy during aerobic respiration. In high concentration, oxygen returns to its beginnings as a toxic molecule and can damage the cells.

The brain is particularly vulnerable to oxidative stress because of several unique characteristics. It has an exceptionally high metabolic demand, consuming nearly 20% of the body’s total oxygen, which in turn leads to substantial generation of reactive oxygen species (ROS). This occurs largely due to the abundance of mitochondria within neuronal cells, where oxidative phosphorylation predominates [5]. The imbalance between pro-oxidants and antioxidant defense causes increased oxidative stress. Mitochondria, as the primary source of ROS, also play a central role in energy production and calcium homeostasis [6,7]. Moreover, the activity of the N-methyl-D-aspartate (NMDA) receptor is crucial not only for normal brain development but also for recovery following cerebral injury [8]. Overstimulation of NMDA receptors can increase mitochondrial ROS production through calcium-mediated cytotoxicity and disrupted mitochondrial calcium handling [9].

Under normal conditions, brain tissue is continuously exposed to ROS and must maintain robust defense mechanisms to counteract oxidative stress. Certain physiological enzymatic reactions also contribute to ROS generation [10]. The brain’s chemical composition further increases its susceptibility. It is rich in unsaturated lipids, which are highly vulnerable to ROS-induced lipid peroxidation [11]. Other macromolecules, including DNA and proteins, are also susceptible to oxidative damage, leading to neuronal dysfunction and neurodegeneration [12,13,14]. Furthermore, the high iron content of the brain, although essential for development and function, can exacerbate oxidative stress under pathological conditions [15].

Despite the high production of ROS from normal metabolic activity and the brain’s vulnerable composition, endogenous antioxidant defenses in the brain are relatively limited. Therefore, maintaining ROS at optimal levels is essential for preserving both structural and functional integrity. Antioxidant strategies in the brain rely on enzymatic defenses, including catalase (CAT), superoxide dismutase (SOD), and glutathione peroxidase (GPX), as well as small endogenous molecules such as reduced glutathione (GSH) and nicotinamide adenine dinucleotide phosphate (NADPH) [16]. Under stressful conditions, such as brain injury, these protective mechanisms can be overwhelmed, leading to excessive free radical accumulation and oxidative stress.

After ischemic stroke, damage extends beyond brain tissue to the neuromuscular unit (NMU). Injury to upper motor neurons and disrupted synaptic transmission leads to muscle fiber atrophy. Additionally, the NMU is highly susceptible to ferroptosis and oxidative damage, which further contributes to neuronal injury and muscle degeneration

### 1.2. Ferroptosis-Mediated Oxidative Damage of the Brain Tissue

Nowadays, several iron-related molecular mechanisms have been implicated in brain disorders, although direct causal link with neuronal injury remains unclear. Neuronal iron homeostasis dysregulation has a negative impact on neuronal cell function. Dysregulation of neuronal iron homeostasis negatively impacts neuronal function, primarily through pathways involving oxidative stress and mitochondrial dysfunction, including reduced adenosine triphosphate (ATP) production and elevated ROS levels. The blood–brain barrier (BBB) serves as the primary regulatory checkpoint for cerebral iron load. Iron predominantly crosses the BBB via transferrin-mediated transport, although alternative carriers such as albumin, citrate, and ATP can also facilitate its entry [17].

Intracellular iron overload promotes excessive generation of hydroxyl radicals (HO·) through the sequential Haber–Weiss and Fenton reactions. This iron-dependent mechanism, termed ferroptosis by Dixon and colleagues, is mechanistically and biochemically distinct from other forms of cell death such as apoptosis [18]. Ferroptosis is characterized by dysregulated iron metabolism, ROS accumulation, and lipid peroxidation in membranes enriched with polyunsaturated fatty acids. Advances in minimally invasive imaging, particularly magnetic resonance imaging (MRI), have provided in vivo evidence that iron dysregulation is a hallmark of neurodegeneration. These studies underscore multiple pathological pathways, including disruption of membrane integrity, proteomic alterations, and mitochondrial dysfunction [15].

Iron is essential for brain and muscle physiology, supporting mitochondrial function, myelin and neurotransmitters synthesis, and muscle metabolism. After stroke, ischemia-reperfusion injury induces mitochondrial dysfunction, oxidative stress, and iron dysregulation, thereby promoting ferroptosis. In its ferrous (Fe^2+^) and ferric (Fe^3+^) forms, iron is indispensable for electron transfer reaction and oxygen transport. However, excess free ferrous iron catalyzes the Fenton reaction, generating reactive oxygen species (ROS) when iron homeostasis fails [19]. Iron overload in the brain—resulting from cell death or hemorrhage—together with downregulation of antioxidant defenses (e.g., GPX4/GSH genes) and upregulation of pro-oxidant enzymes involved in lipid metabolism (e.g., lipoxygenases), constitutes a major driver of ferroptosis [20]. This process contributes to cell death not only in neurons but also in muscle tissue, thereby exacerbating post-stroke damage. These findings highlight ferroptosis as potential therapeutic target. Preclinical studies suggest that limiting iron-driven lipid peroxidation can reduce infarct size and improve neuronal survival, supporting the potential translational value of these therapies [21,22,23,24]. Iron chelators and ferroptosis inhibitors represent promising therapeutic strategies to improve outcomes in stroke patients by limiting iron-driven lipid peroxidation, reducing reactive oxygen species, and preventing ferroptotic neuronal death; preclinical studies and early-phase clinical trials support their potential translational value in ischemic stroke. For instance, the i-DEF trial demonstrated that deferoxamine treatment in 291 patients with moderate intracerebral hemorrhage was associated with improved functional outcomes (modified Rankin Score—mRS score) at 180 days, suggesting its potential role in limiting iron-driven neurotoxicity [25].

One clinical trial has tested deferoxamine in healthy aging individuals, focusing on cerebrovascular function, and reported it as a potentially effective neuroprotective agent [26]. A subsequent clinical trial evaluated deferoxamine in ischemic stroke patients and reported that it is safe and well-tolerated. Moreover, treatment with deferoxamine was associated with improvements in National Institute of Health Stroke Scale (NIHSS) scores and suggested neuroprotective effects in these patients [27]. To date, no clinical trial data are available for ferroptosis inhibitors. However, conflicting results have been reported regarding the use of iron chelators in stroke [28].

A major limitation of deferoxamine is the insufficient understanding of its precise mechanism of action, coupled with the limited number of clinical trials available. Most trials included small cohorts and heterogeneity of outcome measurements, limiting statistical power and generalizability. Also, optimal timing, dosage, and treatment duration for maximal neuroprotection are still undefined. For future studies, some questions should be addressed: (i) if iron chelators or ferroptosis inhibitors could be combined with standard reperfusion strategies (e.g., rtPA, thrombectomy) or antioxidants for synergistic neuroprotection; (ii) identify biomarkers able to select patients who may benefit most.

### 1.3. Iron Dysregulation in the Neuromuscular Unit (NMU) After Stroke

During the acute phase of ischemic stroke, neuroinflammation is the primary event triggered by necrotic cells in the core area. This inflammatory response drives secondary brain injury by increasing ROS production, promoting excitotoxicity and causing microglial/astrocyte activation. Further activated glial cells release a variety of pro-inflammatory molecules, including cytokines, chemokines, and matrix metalloproteases (MMPs). Neuronal cells and microglia are particularly sensitive to iron dysregulation. Both iron deficiency and overload can impair mitochondrial function and increase oxidative stress [29].

The neurovascular unit (NVU) and neuromuscular unit (NMU) play important roles in maintaining iron homeostasis [21,30,31]. The interplay between brain and muscle is a complex and dynamic process, involving multiple molecular and physiological pathways. In recent decades, research has highlighted that muscle cells are more than “posture and movement” units; they secrete bioactive peptides, including cytokines and interleukins, which help regulate energy homeostasis and immune function [21,32,33]. After stroke, neuromuscular junction (NMJ), NVU, and NMU function are disrupted by neuroinflammation. Ferroptosis contributes to oxidative NMJ degeneration by impairing synaptic stability, presynaptic vesicle release, and acetylcholine receptor function.

The post-stroke recovery period is accompanied by dysfunction of both the NVU [34] and NMU, driven by increased neuroinflammation, oxidative stress, and impaired motor neuron signaling [35]. Muscle metabolism is also affected, as iron overload can trigger ferroptosis and motor neuron death [36]. Furthermore, loss of trophic support to muscles promotes atrophy, which disrupts iron metabolism by decreasing ferritin expression and reducing mitochondrial demand due to diminished motor input [37]. Additionally, myoglobin catabolism contributes to elevated iron levels, further exacerbating oxidative stress and muscle dysfunction [38].

Nowadays, it is well known that pro-oxidant and antioxidant imbalance causes chronic inflammation and disrupts brain homeostasis through microglia/astrocyte activation [39,40]. Significant progress has been made in recent years in understanding the role of exogenous and endogenous antioxidant protective mechanisms. Not only have several different exogenous compounds been identified as major players in regulating brain redox balance, but redox state is also controlled by a complex network of interacting feedback mechanisms that involve neural cell defense mechanisms, intracellular signaling, and neuroprotection.

Accumulation of oxidized DNA and proteins, as well as lipid peroxidation, can activate signaling pathways associated with apoptosis, necrosis, and neuronal cell death, thereby impairing functional recovery after cerebral ischemia [10,41]. Consequently, modulation of redox balance in response to oxidative stress represents a promising strategy to enhance neuroprotection and restore function following injury [42,43]. However, a critical gap remains regarding the optimal timing and type of intervention needed to prevent alterations in the NMU and NMJ, which can lead to shifts in muscle fiber composition from slow-twitch (type I) to fast-twitch (type II) fibers.

### 1.4. Mitochondrial Dysfunction and Lack of Energy After Ischemic Injury

Mitochondrial dysfunction is a major contributor to impaired functional recovery after stroke. Following ischemic injury, mitochondrial dysfunction occurs in two main stages. First, ischemia triggers neuroinflammation through activation of the hypoxia-inducible factor (HIF) signaling pathway. HIF activation is an adaptive response to hypoxia, with both protective and deleterious consequences. The protective effects are partly mediated by HIF-induced transcription of genes involved in angiogenesis, cellular metabolism, and survival, enhancing tissue resilience to ischemic stress. Hypoxia promotes dimerization of oxygen-sensitive HIFα subunits (HIF1α and HIF2α) with HIF1β and inhibits prolyl hydroxylase (PHD)-mediated hydroxylation of HIFα. This prevents recognition by the von Hippel–Lindau (VHL) protein and subsequent degradation. The stabilized HIF dimers act as transcription factors by binding to hypoxia response elements (HREs) in DNA, initiating expression of adaptive genes [44,45]. However, HIF-mediated pathways, including mitophagy, can also contribute to cellular dysfunction by disrupting calcium homeostasis, reducing availability of metabolic substrates, and increasing metabolic waste [45].

In the second stage, following reperfusion, the abrupt restoration of oxygen supply allows the electron transport chain (ETC) to resume function, although dysfunction may persist in this pathway. A key mechanism driving increased ROS production during reperfusion is reverse electron transport (RET), which leads to partial reduction in oxygen [45,46]. The resulting ROS accumulation exacerbates mitochondrial dysfunction by increasing membrane permeability, causing loss of membrane potential, and promoting apoptosis or necrosis. Prolonged or excessive apoptosis further contributes to tissue damage and amplifies neuroinflammation, ultimately impairing functional recovery.

### 1.5. Mitochondrial Dysfunction and Lack of Energy in Muscles After Stroke

Disruption of cerebral blood flow after stroke leads to mitochondrial dysfunction that affects not only the brain but also peripheral tissues, including skeletal muscle. Post-stroke muscle impairment arises from energy failure within muscle cells, resulting initially in acute weakness and, without appropriate intervention, progressing to chronic muscle weakness and loss of motor control. The severity and duration of muscle dysfunction are closely linked to the extent of mitochondrial impairment, as muscle tissue has high energy demands [47,48,49]. Proper mitochondrial function is essential for muscle cell regeneration and protein synthesis—two processes critical for maintaining muscle mass and motor performance. Although mitochondrial dysfunction plays a complex role in post-stroke muscle deterioration, its modulation remains a key therapeutic target to preserve muscle function [45]. Nonetheless, deeper understanding is needed to optimize interventions that effectively control this mechanism.

**Figure 1:**
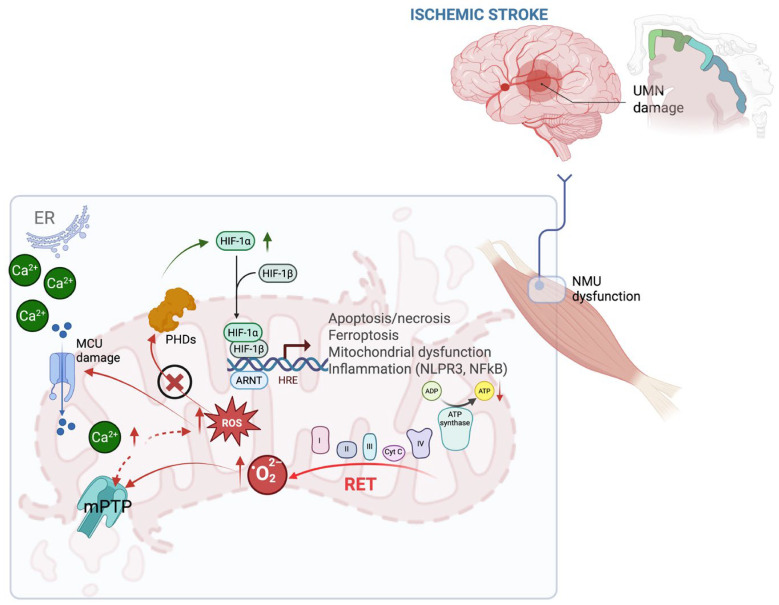
Oxidative stress as a key barrier to functional recovery after ischemic stroke. Brain mirror events: (i) hypoxia triggers mitochondrial dysfunction and activates HIF 1 alpha signalling pathways that act both protectively and through excessive increased ROS production (*ROS* induced mitochondrial damage); (ii) due to increased calcium ions in cytosol and excess of ROS, motor neurons suffer *Ca^2+^*-induced mitochondrial damage, disrupting neuromuscular signalling that contributes to contractile dysfunction, fatigue, and atrophy. Abbreviations: ARNT: aryl hydrocarbon receptor nuclear translocator; ATP: adenosine triphosphate; CoQ: coenzyme Q; Cy c: cytochrome c; ETC: electron transport chain; ER: endoplasmic reticulum; HIF-1α/β: hypoxia-inducible factor 1 alpha/beta; HRE: hypoxia response element; MCU: mitochondrial calcium uniporter; mPTP: mitochondrial permeability transition pore; NF-κB: nuclear factor κB; NMU: neuromotor unit; PHDs: HIF prolyl hydroxylases; RET: reverse electron transport; ROS: reactive oxygen species; UMN: upper motor neuron. (Created in BioRender. b, a. (2025) https://BioRender.com/8rjylth (accessed on 7 July 2025)).

During the hypoxia stage of ischemic stroke, muscle fibers are deprived of oxygen and essential nutrients. Mitochondrial dysfunction at this stage results in energy failure, leading to muscle weakness and loss of motor control, which may progress to muscle atrophy if hypoxia or ischemia persist. Additionally, calcium overload and excessive ROS production disrupt mitochondrial membrane permeability, further contributing to muscle cell death during ischemia-reperfusion injury [45,50,51], see Figure 1.

Mitochondrial dynamics are also disrupted following ischemic stroke, impairing quality control of mitochondrial function. Cellular stress overactivates mitochondrial fission, resulting in fragmented mitochondria and reduced mitophagy, which further exacerbate muscle cell damage [45].

### 1.6. Antioxidative Strategies with an Impact on Functional Recovery Targeting NMU

Post-stroke functional recovery is a complex process involving enhanced neuroplasticity, muscle remodeling, and neuromuscular junction (NMJ) function. Effective interventions must address all these processes to optimize recovery. Numerous studies have focused on strategies to promote cortical reorganization after stroke, including axonal synaptogenesis and sprouting, using techniques such as transcranial magnetic stimulation or transcranial direct current stimulation [52,53]. Additionally, functional electrical stimulation (FES) and neuromuscular electrical stimulation have been employed to support NMJ repair and improve motor unit function [54].

Protection of the post-stroke neuromuscular unit (NMU) primarily follows two directions: preserving NMJ function and preventing muscle atrophy and fiber-type shifts. Over the past decades, many interventions targeting these processes have been evaluated in clinical trials. Given the central role of mitochondria in ischemia/reperfusion injury, research has increasingly focused on identifying interventions that restore mitochondrial function at the optimal time. These approaches include antioxidative strategies, mitochondrial membrane stabilizers (e.g., mPTP inhibitors) [45,55], and even transplantation of new mitochondria [45]. In Table 1 we summarize available antioxidant strategies that target mitochondria and neutralize excess of ROS during ischemia/reperfusion injury.

Antioxidants play a vital role in protecting mitochondria from oxidative stress. Each antioxidant exerts its action through distinct mechanisms that converge on the reduction in oxidative stress and preservation of cellular integrity after stroke. While general antioxidants such as CoQ10, but also mitochondria-targeted antioxidant MitoQ, stabilize ETC function, reducing mitochondrial ROS production and preserving membrane potential, CoQ10 (ubiquinone) stabilizes ETC function by ensuring continuous electron flow, thereby reducing electron linkage that limit ROS production. Also, reduced form of CoQ10 (ubiquinol) prevents lipid peroxidation by scavenging lipid peroxyl radicals, thereby protecting mitochondrial membranes and maintaining cellular integrity [65]. Mitochondrial-targeted antioxidants such as MitoQ are engineered compounds that accumulate into the mitochondria, scavenge mitochondrial ROS, preserve ATP production, and protect against apoptosis and ferroptosis [66]. NAC compound, a precursor for glutathione biosynthesis acts by enhancing intracellular redox buffering and detoxifies hydrogen peroxide and lipid peroxides [67].

ALA acts both as a mitochondrial cofactor and as a potent antioxidant. It supports energy metabolism through its role in pyruvate dehydrogenase and α-ketoglutarate dehydrogenase complexes, while its reduced form (dihydrolipoic acid) scavenges ROS and regenerates other antioxidants such as vitamin C, vitamin E, and glutathione. In addition, ALA chelates redox-active metals (e.g., iron and copper) and exerts anti-inflammatory effects, thereby contributing to neuroprotection in the post-stroke setting [68]. Other compounds from indoleamine class, act as both direct and indirect scavengers. Melatonin directly scavenges ROS and reactive nitrogen species, while also upregulating antioxidant enzymes such as superoxide dismutase and glutathione peroxidase. Indirect, melatonin stabilizes mitochondrial function by preserving membrane potential, reducing cytochrome c release, and modulating apoptotic signaling, thereby supporting neuronal survival after stroke [69].

However, it remains uncertain whether antioxidant molecules are beneficial during the early stages of ischemic stroke or whether they improve long-term functional outcomes in stroke survivors. Consistent with this, recent clinical trial data on the use of antioxidants during acute stroke treatment or rehabilitation have yielded conflicting results. Some human studies suggest that antioxidants offer neuroprotective effects by reducing oxidative stress [13]. Other trials have shown no significant benefit in using antioxidants for stroke recovery in clinical trial [56,57,62]. Although many antioxidant molecules have successfully improved motor function and stroke recovery in preclinical studies, their translation to clinical practice has largely failed, highlighting a critical gap between bench and bedside. Preclinical studies have been highly heterogeneous, ranging from dietary interventions (e.g., vitamins, nutritional supplements, high-energy protein diets) to mechanistic or pharmacological approaches targeting mitochondrial function and mitigating oxidative stress. In contrast, clinical studies investigating antioxidative strategies specifically aimed at mitochondrial dysfunction remain scarce, despite their considerable future potential. Notable interventions to control global and mitochondrial oxidative stress and enhance post-stroke motor function include general antioxidants, mitochondria-targeted antioxidants, and specific strategies such as personalized exercise programs or gene replacement therapies [70].

Over the last 10 years our search in available databases identified ten relevant clinical trials and one metanalysis, which included nine randomized clinical trials and four cohort studies, summarized in Table 1. The relevant clinical trial data was searched in PubMed, Web of Science, and Clinicaltrials.gov database.

Most of the clinical trials are focused on acute phase stroke (first 24 h) with follow-up between 14 days and 30 days post-stroke to test whether early antioxidant intervention can change the functional outcome trajectories of stroke survivors and can improve the quality of life [57,60,61]. However, the inclusion criteria were often too restrictive for stroke patients, as they did not take other comorbidities into account.

We remarked that the studies are heterogenous and included a broad range of antioxidants, such as high-dose vitamin C, mixed vitamin combination, antioxidant cocktails, or endogenous antioxidant boosters (e.g., NAC as a glutathione replenisher) [57,58,59]. Also, antioxidants that are modified to go inside the mitochondria and act as mitochondrial ROS scavengers or neurohormones that have antioxidant properties (e.g., melatonin) were tested in stroke patients that received standard therapy [60].

The main outcome measurements for motor and functional recovery outcomes were assessed using available scales (e.g., National Institutes of Health Stroke Scale—NIHSS for neurological deficits, mRS for functional independence, and Barthel Index for daily living activity) [57,60,61]. Less used were motor-specific scales (e.g., Fugl-Meyer Motor Assessment or gait speed scale).

In addition, oxidative stress biomarkers frequently measured in clinical trials to evaluate the response to antioxidant interventions were malondialdehyde (MDA) and superoxide dismutase (SOD) activity [56,57,58]. Notable studies report novel antioxidant drugs, such as endaravone and endaravone, combined with a terpene compound that may increase its actions.

Endaravone is a compound with a benzothiazine ring structure with a carbonyl group and a free radical-scavenging moiety that makes it a powerful antioxidant. A recent meta-analysis that collected and pooled data from nine available randomised clinical trials (on a cohort of 2000 stroke survivors) concluded that endaravone together with rtPA has a clear benefit on neurological recovery in acute phases, but with no clear benefit for long-term functional recovery [61]. The major strengths of this analysis are the large cohort and well-established intervention and outcome measurements.

Recently, Kashbour et al. [71] stated in their systematic review and meta-analysis of five available RCTs (total of a 2535 stroke patient cohort) that using combined antioxidant/anti-inflammatory therapy (endaravone and dexborneol) there was a small but significant improvement in neurologic scores by 1 month. In this study the pool effect showed a statistical robustness (*p* < 0.0001), indicating that combined antioxidant–anti-inflammatory therapy can yield a beneficial effect that can be translated into a better motor and functional recovery after stroke. It is one of the most robust evidence-based antioxidant interventions in the acute phase of stroke, up to date.

Other individual results for specific antioxidant interventions display limited or no clear benefit. Among this, a melatonin pilot study reports a positive trend on the motor and functional recovery of stroke survivors [60]. This is a small cohort pilot study on the Iranian population, but due to its safety and low cost, it supports further studies on large multiethnic cohorts.

On the other side, studies on NAC, vitamins, and other antioxidant supplements (e.g., ALA) showed limited or no clinical benefits. ALA is a powerful antioxidant that in animal models was successful acting through nuclear receptor factor 2 (NRF2) pathway activation, but the clinical evidence-based results are sparse. However, short-chain fatty acids such as ALA, lack clear data on motor function and pending large cohort studies.

Similar with ALA and melatonin, CoQ10 studies reveal no or limited functional improvement. First, Ramezani and colleagues report that after 300 mg/day of CoQ10 supplement, a significant improvement in neurological and cognitive recovery was obtained, but no significant difference in disability outcome and oxidative stress markers was observed [57]. The authors explain these findings by the need for dose and time adjustments to observe biochemical and clinical responses. Five years later, a recent study by Mojaver and colleagues performed the same study using a higher dose of CoQ10 (600 mg/day for 4 weeks) and reported a significant increase in serum MDA, SOD level, and BDNF level [56]. This study suggests that CoQ10 can decrease oxidative stress, improve antioxidant capacity and support neuronal recovery in short-term follow-up, but no measurements of motor or functional outcomes were reported. In this light, a larger clinical trial is needed to gain evidence-based strategies that can translate this molecule into clinical use.

Cyclosporin A, is an immunosuppressive agent that acts as a neuroprotector by inhibiting mPTP, preventing mitochondrial damage, reducing ROS production, and preventing neuronal cell death. It was reported as a promising tool in preclinical models of stroke [72,73]. Similar to Cyclosporin A, MitoQ is a lipophilic agent (cationic antioxidant) that can penetrate mitochondria and act as an ROS scavenger [74]. Its structure is formed by ubiquinone and a lipophilic part that allow it to pass the mitochondrial membrane and exert its action. In preclinical models of ischemic stroke (e.g., like middle cerebral artery occlusion) and ischemia-reperfusion injury, MitoQ was reported to conserve mitochondrial membrane integrity and prevent cell damage [75].

Other current strategies that target mitochondria and direct or indirect ROS production are currently being tested. Such intervention targets mitochondria using multiple strategies. Some of these are aimed to maintain mitochondrial integrity using mitochondrial dynamic modulators that limit mitochondrial fission (e.g., Drp1 protein inhibitors) or using gene therapies that promote mitochondrial fusion (e.g., melatonin upregulates Opa 1gene expression and restores Mfn2 gene expression) [55]. Other interventions are aimed at replacing damaged mitochondria by exogenous mitochondrial transplantation [45,55], or upregulating protective genes by editing mitochondrial DNA (mtDNA) [45].

### 1.7. Antioxidant Versus Mitochondrial Transplantation in Post-Stroke Rehabilitation

An important challenge in post-stroke recovery is the excess production of ROS, mitochondrial damage, and the consequent energy failure. Over the last decade, numerous compounds such as vitamins, polyphenols, and more specific mitochondria-targeted agents (see Table 1) have been investigated as a strategy to reduce oxidative stress and stabilise the mitochondrial membrane. However, both general and targeted antioxidants have shown only partial efficacy in restoring mitochondrial bioenergetics, which may explain the limited success of their clinical translation. This suggests that more effective strategies must extend beyond ROS scavenging and membrane stabilisation. Approaches such as enhancing mitochondrial biogenesis, metabolic reprogramming, or even mitochondrial transplantation may provide a more robust restoration of mitochondrial function and improve cellular energy balance [55,76].

**Figure 2:**
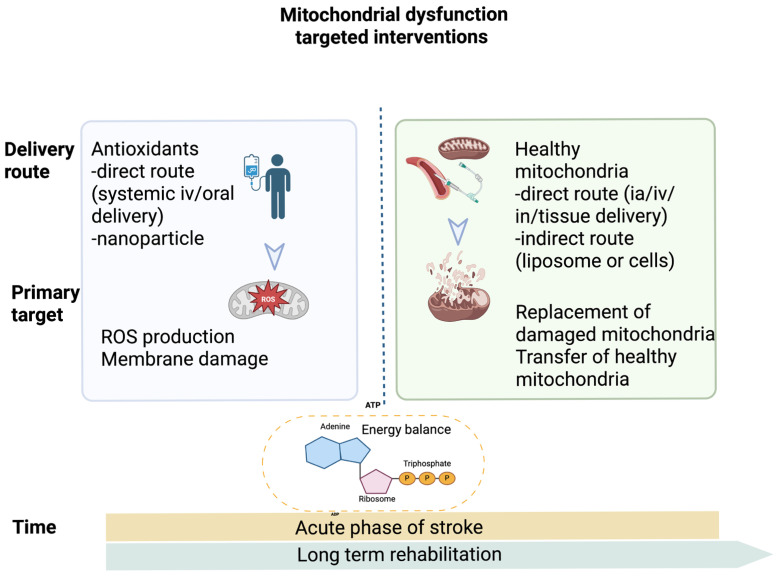
Generic antioxidants versus mitochondria transplant in stroke recovery. Route of delivery for isolated healthy mitochondria: (i) direct route—systemic delivery, intratissue delivery, or intranasal delivery; (ii) indirect route by inclusion in biocompatible carriers (nanoparticle or cells). Abbreviations: ATP: adenosine triphosphate; intraarterial delivery (ia); intravenous delivery (iv); intranasal delivery (in); ROS: reactive oxygen species; (Created in BioRender. b, a. (2025) https://BioRender.com/vju65hf (accessed on 20 August 2025)).

In this context, early preclinical studies on mitochondrial transplantation have reported encouraging results in restoring energy balance and promoting neuroplasticity, underscoring its therapeutic potential in post-stroke recovery [45,76,77]. From a clinical perspective, both generic antioxidants and mitochondrial transplantation present advantages and limitations depending on delivery route, timing, and safety consideration (see Figure 2).

Mitochondrial transplantations currently investigated for more effective route of delivery include direct systemic administration (intraarterial, intravenous, or intranasal delivery) or indirect approaches such as encapsulation in liposomes or extracellular vesicles (EVs), or delivery of bioengineered mitochondria [78,79]. A recent review by Kubat and colleagues summarized current transplantation methods, highlighting their main limitation and the future perspectives. Notably, the authors concluded that co-delivery of mitochondria with protective agents, such as antioxidants, may improve mitochondrial viability within the host cellular environment [80].

At present, conflicting results have been reported regarding the relationship between mitochondrial transplantation and intracellular ROS levels. While some studies suggest that mitochondria transfer can exacerbate oxidative stress, other report beneficial effects [81,82,83]. Despite these discrepancies, control of oxidative stress remains a critical factor for the success of mitochondrial transplantation.

Additional limitations that must be addressed include immunogenic potential of transplanted mitochondria, their limited viability, and challenges of integration into the recipient cellular network. Thus, although mitochondrial transplantation holds considerable promise for post-stroke recovery, its optimisation requires further investigation. Key open questions include whether co-transplantation with generic antioxidants or pre-treatment with ROS-modulating interventions (e.g., antioxidants, physical exercise training) could better prepare the recipient microenvironment, thereby enhancing mitochondrial survival, integration, and neuroplasticity. Compared with generic antioxidants (e.g., endaravone), which are already approved for clinical use in acute ischemic stroke in certain regions, no standardised protocols for mitochondrial transplantation are currently available. To date, preclinical investigation and early phase clinical trial are ongoing (e.g., NCT04998357), and additional evidence-based results are needed before its implementation in clinical practice.

## 2. Clinical Impact and Future Perspective

The discussion of the antioxidant strategies in the context of post-stroke recovery and its potential as a therapeutic target highlights several limitations and future perspectives. The use of antioxidants in stroke has yielded heterogenous results in clinical trials, likely due to multiple factors that require further consideration. For example, edaravone and the combination of edaravone/dexborneol have shown promising neuroprotective effects in acute ischemic stroke; however, it is important to note that the supporting clinical evidence is predominantly derived from Asian populations. This geographic concentration of data may limit the generalizability of the findings to other populations, due to potential differences in genetics, stroke subtype prevalence, comorbidities, and standard of care. Future multicenter trials including more diverse populations are therefore essential to validate the efficacy and safety of these interventions globally. Factors such as the timing of delivery and safety concerns, stroke type and severity, as well as the treatment window (acute versus long-term phases) play important roles in determining therapeutic efficacy. Beyond these biological considerations, study design issues including small cohort, heterogeneity in study design, limited follow-up duration, and low consistency of clinical outcome further contributed to the inconsistency of clinical findings. Such limitation underscores the need for more rigorously designed clinical trials before firm conclusions can be drawn.

Nonetheless, certain antioxidant compounds remain promising. Immunosuppressive agents (e.g., cyclosporin A), for example, have shown beneficial effects in preclinical stroke models by reducing both oxidative stress and neuroinflammation. This dual mechanism has demonstrated beneficial effects on post-stroke tissue repair and functional recovery, though their translational potential will depend on replication in larger randomized controlled clinical trials (see Table 1). Likewise, mitochondria-targeted antioxidants such as MitoQ are still in early stages of clinical investigation. Future studies will need to clarify its long-term efficacy, effective dosing, and its clinical impact on post-stroke recovery. In addition to mechanistic issues, the incomplete understanding of the pathogenic mechanisms underlying mitochondria-targeted antioxidants also limits the translation potential for post-stroke recovery.

Overall, the clinical impact of antioxidants in post-stroke remains a subject of ongoing debate. Although preclinical models consistently demonstrate neuroprotection and functional improvement, translation into meaningful clinical outcomes has been inconsistent. There are several key points based on current findings that deserve future attention, such as neuroprotective effect in the acute phase or its potential for recovery in long-term post-stroke survivors (e.g., improving motor functions). For long-term recovery, combined interventions such as molecular tools combined with physical and cognitive therapies can enhance recovery and achieve better clinical outcomes. Although, future clinical research on antioxidant therapies in stroke should explore combination strategies that target multiple pathogenic pathways, such as general and mitochondria-targeted antioxidants or iron chelators. Patient stratification using biomarkers of oxidative stress or ferroptosis may improve therapeutic precision, while multicenter trials including diverse populations are essential to enhance generalizability. Randomized, double-blind, placebo-controlled designs with clearly defined functional endpoints will strengthen the translational potential of these interventions. Together, these strategies can more effectively evaluate and harness antioxidant targeted therapies for post-stroke recovery

Currently, future therapies must consider individuals and their potential response to intervention and move forward to personalized medicine. All of these can lead to more successful therapies.

## 3. Conclusions

Mitochondrial dysfunction in stroke contributes not only to brain tissue damage but also to secondary muscle damage through mechanisms such as ATP production failure, excessive ROS generation, calcium dysregulation, and impaired mitochondrial dynamics. These processes lead to muscle weakness, atrophy, and neurogenic muscle tissue damage.

Emerging mitochondria-targeted therapies hold promise for enhancing recovery and supporting muscle repair in stroke patients. General antioxidants mitigate cellular and tissue injury by neutralizing ROS, limiting inflammation, and partially improving energy metabolism. However, the therapeutic effect of both antioxidants and mitochondrial transplantation remains incomplete and requires further optimization. Key rehabilitation research priorities include the development of relevant outcome measurements that capture fatigue, endurance, gait autonomy, and rehabilitation compliance. The antioxidants target oxidative stress, but this is strongly related to neuroinflammation. In this light, antioxidant therapy must be considered as a piece of the puzzle rather than a “stand alone” strategy for neurorecovery. Furthermore, upcoming clinical trials should be designed to test the synergistic effect of ROS-modulating compounds, mitochondrial transplantation, and structured physical rehabilitation program, with the aim of achieving a more comprehensive restoration of mitochondrial health and, ultimately, improving long-term rehabilitation outcomes.

## Figures and Tables

**Table 1:** Emerging antioxidant strategies in recent clinical trials aimed to improve mitochondrial dysfunction and functional recovery.

Intervention Type	Compounds/Approach	Mechanism/Target	Study Design	Key outcome	Statistical Significance	Clinical Relevance
General antioxidant	CoQ10 [56]	Support ETC function	Double-blind RCT, *n* = 50, 600 mg/day × 1 week, acute stroke (<24 h), 1-week follow-up	↓ serum MDA, ↓ IL6, ↑ BDNF and SOD level	Yes (*p* < 0.05)	Potential neuroprotective benefits
	Vit. E + Vit. C [57]	ROS neutralization, lipid peroxidation prevention	Double-blind, RCT, *n* = 60, 300 mg/day x 4 weeks, acute stroke (<24 h), 4-week follow-up	improved NIHSS and MMSE score; no significant changes in MDA, SOD, GFAP	Yes (*p* = 0.05 for NIHSS; *p* = 0.03 for MMSE)	Functional benefit, unclear mechanistic impact
	NAC [58,59]	Glutathione precursor	Double-blind RCT, *n* = 24, 14 days in acute phase (<12 h)	↓ plasma MDA and CRP; ↑ TAC	Yes (*p* < 0.003) for TAC and *p* < 0.002 for MDA	Possible adjuvant benefit
			RCT, *n* = 40, NAC + rtPA within 4.5 h after stroke,	Ongoing Phase II (NCT04918719)	-	No results
	Melatonin [60].	Free radical scavenger; antioxidant targeted at the mitochondria	Double-blind RCT, *n* = 65, 20 mg/day × 5 days, <24 h post-stroke, 90 d follow-up	improved NIHSS and MMSE score	Yes	Potential neuroprotective benefits
	Endaravone [61]	Free radical scavenger	9 RCT + 4 cohort studies, *n* = 2102, Endaravone + rtPA, <24 h post-stroke; short-term follow-up	improved Barthel index, NIHSS	Yes (*p* < 0.001 for Barthel index; *p* = 0.003 for NIHSS	Neuroprotective benefits
*Mitochondria-targeted antioxidants*						
	MitoQ	Scavenging mitochondrial ROS	1 × 80 mg of Mito Q, 45 min follow-up	Ongoing Phase III stroke trials NCT06930638	No available data	Clinical data pending
	ALA [62]	Scavenging mitochondrial ROS, enhancing endogenous antioxidant capacity	RCT, *n* = 67, 1200 mg/day × 3 weeks	↓ TNF-α, ↓ IL6 No significant for antioxidant markers,	Mixed	Anti-inflammatory effect, unclear functional relevance
			ALA + rtPA within 6 h	Ongoing Phase III (NCT04041167)	No available data	Clinical data pending
*Exercise-targeted mitochondrial biogenesis*	Aerobic Exercise [63]	Activates PGC-1α → ↑ mitochondrial biogenesis, ↑ antioxidant capacity	Blinded RCT, 36 sessions/12 weeks, subacute/chronic stroke	Improved cognitive function	yes	Relevant cognitive benefit
*mPTP-targeted antioxidants*						
	Cyclosporin A [64]	Inhibits cyclophilin D, prevents mPTP opening	Phase II, ICT, n = 126, 2 mg/kg bolus within 15 min of thrombolysis	Did not significantly reduce infarct size (NCT01527240)	No	No evidence-based clinical benefit up to date

Coenzyme Q10 = CoQ10; ETC = electron transport chain; RCT = randomized controlled trial; MDA = malondialdehyde; IL6 = Interleukin 6; BDNF = brain derived neurotrophic factor; SOD = superoxide dismutase; vit E = alpha-tocopherol; vit C = ascorbic acid; NAC = N-acetylcysteine; NIHSS = National Institutes of Health Stroke Scale; MMSE = Mini-Mental State Examination; GFAP = Glial Fibrillary Acidic Protein; TAC = Total Antioxidant Capacity; CRP = C Reactive Protein; rtPA = tissue plasminogen activator (alteplase); Mitoquinone = MitoQ; ALA = Alpha-lipoic acid; TNF-α = tumour necrosis factor alpha; PGC-1α—Peroxisome Proliferator-Activated Receptor Gamma Coactivator 1-Alpha; mPTP—mitochondrial permeability transition pore; ICT = interventional clinical trial.

## Data Availability

Not applicable.

## Abbreviations

The following abbreviations are used in this manuscript:

ALA	Alpha-lipoic acid
ARNT	Aryl hydrocarbon receptor nuclear translocator
ATP	Adenosine triphosphate
BBB	Brain blood barrier
BDNF	Brain derived neurotrophic factor
CoQ:	Coenzyme Q
CoQ10	Coenzyme Q10
CRP	C Reactive Protein
Cy c	Cytochrome c
DNA	Deoxynucleic acid
Drp1	Dynamin-related protein 1
ER	Endoplasmic reticulum
ETC	Electron transport chain
EVs	Extracellular vesicles
Fe2+	Ferrous form of iron
Fe3+	Ferric form of iron
FES	Functional electrical stimulation
GFAP	Glial fibrillary acidic protein
GPX4	Glutathione peroxidase 4
GSH	Reduced form of glutathione
HIF	Hypoxia-inducible factor
HO	Hydroxyl radical
HREs	Hypoxia response element
ICT	Interventional clinical trial
IL6	Interleukin 6
IV	Intravenous
MCU	Mitochondrial calcium uniporter
MDA	Malondialdehyde
MitoQ	Mitoquinone
MMPs	Matrix metalloproteases
MMSE	Mini-Mental State Examination
mPTP	Mitochondrial permeability transition pore
MRI	Magnetic resonance imaging
mRS	Modified Rankin Scale
mtDNA	Mitochondrial DNA
NAC	N-acetylcysteine
NADPH	Nicotinamide adenine dinucleotide phosphate (reduced form)
NF-κB:	Nuclear factor κB
NIHSS	National Institutes of Health Stroke Scale
NMDA	N-methyl-D-aspartate receptor
NMJ	Neuromuscular junction
NMU	Neuromotor unit
NRF2	Nuclear receptor factor 2
NVU	Neurovascular unit
Opa 1	OPA1 mitochondrial dynamin-like GTPase
PGC-1α	Proliferator-activated receptor-gamma coactivator 1-alpha
PHDs	HIF prolyl hydroxylases
RCT	Randomized controlled trial
RET	Reverse electron transport
RNA	Ribonucleic acid
ROS	Reactive oxygen species
rtPA	Recombined tissue plasminogen activator
SOD	Superoxide dismutase
TAC	Total Antioxidant Capacity
TNF alpha	Tumour necrosis factor alpha
VHL	von Hippel Lindau
vit C	Ascorbic acid
vit E	Alpha-tocopherol
